# Psychosomatic symptom profiles and associated factors of clinically significant anxiety symptoms among breast cancer patients receiving chemotherapy: a multicenter cross-sectional study in China

**DOI:** 10.3389/fonc.2026.1858680

**Published:** 2026-09-21

**Authors:** Qiong Zhang, Qiqi Sun, Zhifeng Xiong, Hongqiao Fan, Junpeng Chen, Yuan Zhou, Gang Lyu, Xiaohua Pei, Yingyi Fan

**Affiliations:** 1 Department of Breast, The Third Affiliated Hospital of Beijing University of Chinese Medicine, Beijing, China; 2 Department of Breast, Chongqing Hospital of Traditional Chinese Medicine, Chongqing, China; 3 Department of Plastic Surgery, The First Affiliated Hospital of Hunan University of Chinese Medicine, Changsha, China; 4 Department of Breast Surgery, Xiamen Hospital of Traditional Chinese Medicine, Xiamen, China; 5 Department of Breast and Thyroid Surgery, Shaoyang Central Hospital, Shaoyang, China

**Keywords:** associated factors, breast cancer, chemotherapy, clinically significant anxiety symptoms, cross-sectional study

## Abstract

**Background:**

Chemotherapy is a cornerstone of breast cancer treatment, but chemotherapy-induced adverse events and psychosocial factors often impair patients’ quality of life and diminish its clinical benefit.

**Objective:**

To summarize the characteristics of psychosomatic symptoms during chemotherapy for breast cancer and identify associated factors for clinically significant anxiety symptoms.

**Methods:**

A multicenter cross-sectional study was conducted in four hospitals in China. Basic clinical data and medical history were collected for all participants. The 7-item Generalized Anxiety Disorder Scale (GAD-7) was adopted to evaluate clinically significant anxiety symptoms. The MD Anderson Symptom Inventory (MDASI) and the Symptom Assessment Scale for Breast Cancer Patients Undergoing Chemotherapy were used to collect and assess high-frequency psychosomatic distress. The Social Support Rating Scale (SSRS) measured objective support, subjective support, and utilization of social support, and the Resilience Scale Specific to Cancer (RS-SC) was used to evaluate psychological resilience. Univariate and multivariate binary logistic regression analyses were performed to screen associated factors for clinically significant anxiety symptoms, and Spearman’s rank-order correlation was used to explore correlations between high-frequency psychosomatic symptoms and anxiety.

**Results:**

A total of 391 breast cancer patients undergoing chemotherapy were enrolled in this study. The total anxiety incidence was 43.99%, including mild (28.64%), moderate (12.53%), and severe anxiety (2.82%). Binary logistic regression analysis identified clinical stage and cumulative chemotherapy cycles as independent associated factors for anxiety in breast cancer patients during chemotherapy (all *P* < 0.05). Conversely, age, Karnofsky Performance Status (KPS) score, and mental resilience were confirmed as factors associated with lower anxiety symptoms for anxiety in this population (all *P* < 0.05). Spearman’s rank correlation analysis revealed that anxiety was positively correlated with difficulty initiating sleep (*ρ* = 0.358, *P* < 0.01), fatigue (*ρ* = 0.322, *P* < 0.01), and nausea (*ρ* = 0.366, *P* < 0.01).

**Conclusion:**

Observational findings from this study identify several characteristics associated with clinically significant anxiety among breast cancer patients receiving chemotherapy: younger age, low psychological resilience, poor KPS performance status, advanced clinical stage, and longer cumulative chemotherapy cycles. Difficulty initiating sleep, fatigue, and nausea show correlations with anxiety symptoms in this patient population. These findings highlight that physical symptom assessment and psychological screening should not be separated during clinical care for breast cancer patients undergoing chemotherapy, and provide observational evidence for developing integrated supportive care strategies in real-world oncology practice.

**Clinical Trial Registration:**

https://www.chictr.org.cn/, identifier ChiCTR2400086704.

## 1 Introduction

In October 2025, global cancer statistics published in *The Lancet* showed that breast cancer ranked first in incidence and fourth in cancer-related mortality (1). Some studies have proposed an association between chronic psychological stress, including anxiety, depression, and traumatic life events, and breast cancer risk, indicating possible bidirectional adverse interactions between psychological distress and breast-related disease processes (2). Chronic psychological stress has been verified to promote the occurrence, proliferation, invasion, and metastasis of breast cancer through neuroendocrine and immune pathways (3). In turn, breast cancer itself can activate microglia and inhibit astrocyte function in the brain, thereby inducing emotional disorders (4). Therefore, regulating the psychological and mental status of patients with breast cancer represents an important issue in the fields of cancer neuroscience and psychosomatic oncology.

Chemotherapy is a mainstay treatment for breast cancer. Psychological distress stemming from cancer diagnosis and treatment can impair patients’ quality of life for decades (5). Anxiety and depression are not transient states but may persist across the disease trajectory; they can compromise treatment adherence, increase recurrence likelihood, and even negatively influence survival outcomes (6). Chemotherapy itself may further aggravate anxiety symptoms, with epidemiological data reporting anxiety prevalence up to 56% among breast cancer patients receiving chemotherapy (7). From the perspective of the biopsychosocial model (8), anxiety symptoms among breast cancer patients undergoing chemotherapy arise from multiple interacting domains. Guided by this theoretical framework together with contemporary psycho-oncology evidence (9), the present study selected variables across five conceptual domains: individual socio-demographic characteristics, disease related clinical factors, chemotherapy associated psychosomatic symptom burden, social support, and psychological resilience. Individual socio-demographic features may shape baseline vulnerability to cancer related distress; disease related factors can operate as objective stressors eliciting negative emotions; chemotherapy driven somatic discomfort may amplify psychological distress; social support serves as an external stress buffering resource; and psychological resilience represents an internal coping resource regulating patients’ adaptation to cancer related stress. Although anxiety among breast cancer patients has been studied, anxiety specifically during chemotherapy remains under investigated, and evidence regarding its associated factors in this clinical setting remains incomplete.

Accordingly, this multicenter cross-sectional study aimed to characterize psychosomatic symptom profiles and explore associated factors for clinically significant anxiety symptoms among breast cancer patients during chemotherapy.

## 2 Methods

### 2.1 Study design

This clinical study adopted a multicenter, cross-sectional research design. Enrolled patients were divided into two groups: the anxiety symptoms group and the non-anxiety symptoms group. Clinical cases were recruited from patients receiving adjuvant chemotherapy for breast cancer at Xiamen Hospital of Traditional Chinese Medicine, Chongqing Hospital of Traditional Chinese Medicine, The First Affiliated Hospital of Hunan University of Chinese Medicine, and Shaoyang Central Hospital. The screening and enrollment period was from January 2024 to July 2025.

### 2.2 Patients

#### 2.2.1 Diagnostic criteria

1. Breast cancer: based on the 2023 NCCN Clinical Practice Guidelines in Oncology: Breast Cancer (Version 4) (10), and in combination with preoperative puncture biopsy or postoperative pathology clearly defined as breast cancer.
2. Clinically significant anxiety symptoms: Defined as a score of > 4 on the GAD-7 (11).

#### 2.2.2 Inclusion criteria

1. Female, aged 18–80 years;
2. Patients who have received adjuvant chemotherapy in accordance with the NCCN Guidelines, and achieved a disease status of no worse than stable disease (SD) after completing 2–4 cycles of chemotherapy to enter the maintenance treatment phase;
3. Patients with adequate capacity for comprehension and expression, who voluntarily participate in this study and provide written informed consent by signing the informed consent form.

#### 2.2.3 Exclusion criteria

1. Presence of severe aphasia, dysphasia, or deafness resulting in inability to communicate effectively.
2. A history of uncontrolled psychotic disorders complicated by organic mental disorders.
3. Anxiety attacks secondary to other psychiatric disorders or somatic illnesses.
4. Receipt of psychological intervention within the preceding 1 month.

### 2.3 Data gathering

#### 2.3.1 Socio-demographic and related clinical characteristics

Name, age, occupation, educational attainment, monthly household income, marital status, reproductive history, medical insurance type, personal history of psychiatric disorders (including anxiety and depression), comorbidities, physical activity level, place of residence, smoking status, alcohol consumption status, and exposure to negative life events within the preceding week.

#### 2.3.2 Medical history and clinical disease-related information

Surgical history, pathological diagnosis, tumor stage, cumulative number of chemotherapy cycles, and KPS score.

#### 2.3.3 Assessment of clinically significant anxiety symptoms

Clinically significant anxiety symptoms were assessed using the GAD-7 scale: a brief self-report scale for anxiety symptoms that has demonstrated good reliability and validity among Chinese patients with malignant tumors (11). Anxiety symptoms severity was categorized based on the total score, as follows: 0–4 points, no anxiety; 5–9 points, mild anxiety; 10–14 points, moderate anxiety; and ≥ 15 points, severe anxiety.

#### 2.3.4 High-frequency psychosomatic distress during chemotherapy

The MDASI scale and the Symptom Evaluation Scale for Breast Cancer Patients Receiving Chemotherapy were employed to collect data on core psychosomatic symptoms and breast cancer specific symptoms, signs experienced during chemotherapy. Intensity was rated on a 0–10 numerical rating scale, where a score of 0 represented no impact and 10 represented extremely severe impact. Both scales have been validated to exhibit good reliability, validity, and clinical applicability in Chinese patients with malignant tumors (12, 13).

#### 2.3.5 Assessment of social support

Social support was assessed using the SSRS scale. This scale comprises 10 items and three dimensions, namely objective support, subjective support, and support utilization. Specifically, objective support (3 items; higher scores indicate greater objective support) encompasses direct material assistance, group relational ties, and the availability of and participation in social networks. Subjective support (4 items, score range: 8–32 points) includes the emotional sustenance that patients perceive subjectively, such as their emotional experiences and sense of satisfaction derived from being respected, understood, and supported in social contexts. Support utilization (3 items, score range: 3–12 points) reflects how patients make use of available social support resources (14).

Subscale scores were calculated by summing the raw scores of all items within each corresponding dimension. The total SSRS score was obtained by summing the three subscale scores. Higher scores represent better social support status. The scale thus establishes an integrated framework that incorporates both subjective and objective domains, along with the utilization of available support resources. The SSRS scale has been verified to possess good reliability and validity in research involving Chinese cancer patients (14).

#### 2.3.6 Assessment of mental toughness in breast cancer patients

Psychological resilience among breast cancer patients was evaluated using the RS-SC scale for cancer survivors. This scale contains 25 items across five dimensions (disease benefit, support and coping, non-specific resilience components, hope for the future, and meaning of existence). Each item is rated on a 5-point Likert scale (1 = strongly disagree to 5 = strongly agree). Subscale scores are computed as the sum of item scores within each dimension. The total RS-SC score is calculated by summing all 25 item scores; the total score ranges from 25 to 125 points. Higher total scores reflect stronger psychological resilience. This instrument has demonstrated good reliability and validity among Chinese cancer patients (15).

### 2.4 Sample size

The initial sample size was calculated using the population proportion estimation formula for simple random sampling, which was driven by the primary aim to estimate the prevalence of anxiety among breast cancer patients receiving chemotherapy:


$$\text{n}=\frac{{\text{Z}}_{\text{a}/2}^{2}\text{π}(1-\text{π})}{{\text{δ}}^{2}}$$

a = 0.05, Z(a/2) = 1.96, δ = 0.05. Based on the literature the prevalence of anxiety states during chemotherapy for breast cancer is 56% (7), π = 0.56, the required sample size was calculated to be 378.

Multivariable logistic regression was another key analytical objective of this study; however, no formal sample size calculation targeting regression modelling was performed at the study planning stage. Therefore, we conducted a *post-hoc* adequacy assessment using the Events-Per-Variable (EPV) rule after data collection. After exclusion of invalid questionnaires, 391 valid participants were included, among whom 172 participants had anxiety related outcome events. The final multivariate logistic regression model contained 11 predictor variables retained following univariate screening, yielding an EPV value of approximately 15.6. This value exceeds the widely recommended empirical threshold EPV ≥ 10 (16), indicating that the achieved sample size supports stable coefficient estimates for multivariable logistic regression.

### 2.5 Ethics

The study participants or their legal representatives signed the informed consent, which abided by the principles of the Declaration of Helsinki and the regulations on the quality management. The protocol was approved by the ethics committees of the participating hospitals (Number: 2024-K015-02). This trial has been registered at the China Clinical Trial Registration Center (Number: ChiCTR2400086704).

### 2.6 Statistical methods

#### 2.6.1 Missing value treatment

Questionnaires with < 1% missing data variables were considered as qualified questionnaires and did not need to be processed for missing values. Questionnaire data with ≥ 1% missing variables were considered as missing value data. Different methods are used to deal with different types of missing data: when the missing values are basic information, medical history information, and scale scores, the respondents can be contacted by phone to trace the original data to fill in the questionnaire, and for data that cannot be traced back to the original data, linear interpolation can be used to fill in the missing values; when the missing value is a symptom, the symptom has the characteristics of time, degree, etc., and therefore, it can’t be supplemented, and should be excluded from the questionnaire.

#### 2.6.2 Statistical analysis

Data statistics were performed using SPSS 26.0 (IBM, Armonk, NY, USA). Continuous variables were presented as mean ± standard deviation, while categorical variables were reported as constituent ratios. To improve statistical robustness and exclude rare symptoms and signs with low occurrence frequency, symptoms and signs with an occurrence frequency > 70 were defined as high-frequency psychosomatic distress.

Baseline characteristics stratified by each participating center were calculated. For categorical variables, the χ² test was applied when cell counts met statistical assumptions; otherwise, Fisher’s exact test was used. One-way ANOVA was used for between-group comparison of continuous variables. Variables showing significant inter-center differences were included in the multivariate logistic regression model to adjust for multicenter related confounding.

Binary logistic regression was applied to explore associated factors for anxiety related outcomes.

Twenty three candidate socio-demographic, clinical, and psychosocial variables were first individually tested in univariate binary logistic regression analyses. Variables yielding *P* < 0.05 in univariate analyses were subsequently entered into the multivariable logistic regression model. Continuous predictors retained in the multivariable model (age, KPS score, three dimensions of SSRS, and RS-SC total score) were included in their raw form. Categorical predictors were coded as dummy variables; “no” was set as the reference group for binary variables. Ordinal clinical variables (tumor stage, cumulative chemotherapy cycles) used *a priori* predefined binary grouping (≤ IIIA vs > IIIA; ≤ 4 cycles vs > 4 cycles), with lower risk subgroups as reference categories.

Variance inflation factor (VIF) was calculated to assess multicollinearity among predictors within the multivariable model; VIF < 5 was regarded as indicating no obvious multicollinearity. Model performance of the multivariable logistic regression was evaluated via the Hosmer-Lemeshow goodness-of-fit test and Nagelkerke pseudo-R^2^. Odds ratios (OR) together with 95% confidence intervals (95%CI) were reported for regression outputs.

Spearman rank-correlation analysis, using *ρ* to denote the correlation coefficient, was used to examine the correlations between high-frequency psychosomatic symptoms and clinically significant anxiety symptoms. The strength of the correlation was determined according to the absolute value of the correlation coefficient: |*ρ*| < 0.2 was considered a very weak correlation; 0.2 ≤ |*ρ*| < 0.4 a weak correlation; 0.4 ≤ |*ρ*| < 0.6 a moderate correlation; 0.6 ≤ |*ρ*| < 0.8 a strong correlation; and 0.8 ≤ |*ρ*| < 1 a very strong correlation. All statistical tests were two-sided, and a P-value < 0.05 was considered statistically significant.

## 3 Results

### 3.1 Socio-demographic and clinical characteristics of the participants

A total of 410 breast cancer chemotherapy patients were surveyed, out of which 19 questionnaires (4.6%) were excluded due to invalid or missing more than 1%. 391 patients responded effectively with a response rate of 95.37% (**Table 1**). Among these 391 valid participants, 109 patients were enrolled from Xiamen Hospital of Traditional Chinese Medicine, 151 from Chongqing Hospital of Traditional Chinese Medicine, 61 from The First Affiliated Hospital of Hunan University of Chinese Medicine, and 70 from Shaoyang Central Hospital. All participating centers followed unified inclusion and exclusion criteria, data-collection protocols, and adopted identical assessment scales for data acquisition. Baseline patient characteristics across participating centers are compared in **Supplementary Table**.

**Table 1:** Socio-demographic and clinical characteristics of the participants.

Evaluation content	Overall characteristic
Age (mean ± SD, year)	52.19 ± 9.54
Occupation (*n*, [%])	
Mental work	262 (67.01)
Physical work	129 (32.99)
Educational attainment (*n*, [%])	
Bachelor’s degree and above	147 (37.60)
Below bachelor’s degree	244 (62.40)
Household monthly income (*n*, [%])	
≥ 5000 CNY	221 (56.52)
< 5000 CNY	170 (43.48)
Marital status (*n*, [%])	
Married	356 (91.05)
Others: single, widowed, divorced	35 (8.95)
Reproductive history (*n*, [%])	
Yes	349 (89.26)
No	42 (10.74)
Medical insurance (*n*, [%])	
Yes	383 (97.96)
No	8 (2.04)
History of psychiatric disorders such as anxiety and depression (*n*, [%])	
No	381 (97.44)
Yes	10 (2.56)
Comorbidities (*n*, [%])	
No	377 (96.42)
Yes	14 (3.58)
Physical activity (*n*, [%])	
No	374 (95.65)
Yes	17 (4.35)
Place of residence (*n*, [%])	
Urban	326 (83.38)
Rural	65 (16.62)
Smoking (*n*, [%])	
No	386 (98.72)
Yes	5 (1.28)
Alcohol consumption (*n*, [%])	
No	383 (97.95)
Yes	8 (2.05)
Negative life events (in the past week) (*n*, [%])	
Yes	75 (19.18)
No	316 (80.82)
Surgical history (*n*, [%])	
No	83 (21.23)
Yes	308 (78.77)
Pathologic diagnosis (*n*, [%])	
Invasive Ductal Carcinoma	355 (89.20)
Invasive lobular carcinoma	16 (4.69)
Invasive Micropapillary Carcinoma	12 (3.05)
Mucinous Carcinoma	5 (2.11)
Invasive Carcinoma with Neuroendocrine Differentiation	2 (0.47)
Tall Cell Carcinoma with Reversed Polarity	1 (0.24)
Tumor stage (*n*, [%])	
I	68 (18.78)
II	244 (60.80)
III	59 (15.02)
IV	20 (5.40)
Cumulative cycles of chemotherapy (*n*, [%])	
≤ 4 times	340 (86.96)
> 4 times	51 (13.04)
KPS score(mean ± SD)	89.57 ± 2.04
SSRS - Objective Support (mean ± SD)	12.72 ± 3.73
SSRS - Subjective Support (mean ± SD)	22.26 ± 4.47
SSRS - Utilization of Support (mean ± SD)	6.85 ± 2.16
RS-SC score (mean ± SD)	99.08 ± 16.22

Baseline characteristics stratified by the four participating centers are presented in the **Supplementary Table**. Several variables showed significant inter-center differences, including subjective social support, utilization of social support, and cumulative cycles of chemotherapy. To mitigate potential confounding bias introduced by these inter-center imbalances, variables with statistically significant between site discrepancies were incorporated into the subsequent multivariate logistic regression model to account for multicenter related heterogeneity.

### 3.2 High-frequency psychosomatic distress during chemotherapy for breast cancer

A total of 16 symptoms or signs were defined as high-frequency psychosomatic distress. (**Table 2**).

**Table 2:** High-frequency psychosomatic distress during chemotherapy for breast cancer.

Psychosomatic distress	*n*	Rate (%)	Psychosomatic distress	*n*	Rate (%)
Difficulty Initiating Sleep	305	78.01	Leucopenia	249	63.68
Light Sleep	298	76.21	Constipation	185	47.31
Fatigue	297	75.96	Forgetfulness	157	40.15
Early Waking	285	72.89	Xerostomia	98	25.06
Nausea	284	72.63	Hepatic Dysfunction	85	21.74
Alopecia	266	68.03	Paresthesia of the Limbs	81	20.72
Frequent Dreaming	265	67.77	Dizziness	73	18.67
Loss of Appetite	261	66.75	Arthralgia	71	18.16

### 3.3 Prevalence of clinically significant anxiety symptoms

The prevalence of clinically significant anxiety symptoms among breast cancer patients during chemotherapy was 43.99%. Specifically, the proportions of patients with different anxiety severities were as follows: mild anxiety accounted for 28.64%, moderate anxiety for 12.53%, and severe anxiety for 2.82%. (**Table 3**).

**Table 3:** Prevalence of clinically significant anxiety symptoms.

Clinically significant anxiety symptoms	Severity	*n* (%)
No	–	219 (56.01)
Yes	Mildly	112 (28.64)
	Moderately	49 (12.53)
	Severe	11 (2.82)

### 3.4 Analysis of associated factors for prevalence of clinically significant anxiety symptoms in breast cancer patients during chemotherapy

#### 3.4.1 Univariate logistic regression analysis

Univariate logistic regression analysis was performed to assess the associations between Prevalence of clinically significant anxiety symptoms and the following variables: age, occupation, educational attainment, monthly household income, marital status, reproductive history, medical insurance, personal history of psychiatric disorders (including anxiety and depression), comorbidities, physical activity, place of residence, smoking status, alcohol consumption status, exposure to negative life events within the preceding week, surgical history, pathological diagnosis, tumor stage, cumulative number of chemotherapy cycles, KPS score, objective support (SSRS), subjective support (SSRS), support utilization (SSRS), and the RS-SC score.

Following this analysis, 11 variables were identified as candidate factors for subsequent multivariate logistic regression analysis: age, personal history of psychiatric disorders, comorbidities, surgical history, tumor stage, cumulative number of chemotherapy cycles, KPS score, SSRS-objective support, SSRS-subjective support, SSRS-support utilization, and RS-SC score. (**Table 4**; **Figure 1**).

**Table 4:** Univariate logistic regression analysis of associated factors for clinically significant anxiety symptoms during chemotherapy for breast cancer.

Evaluation content	*Beta*	*SE*	*Wald*	*OR*	*P*	95%CI
Age	-0.024	0.011	4.913	0.976	0.027^*^	0.955-0.997
Occupation	0.073	0.219	0.112	1.076	0.737	0.701-1.652
Educational attainment	0.147	0.210	0.492	1.159	0.483	0.768-1.749
Monthly household income	0.018	0.224	0.007	1.018	0.935	0.657-1.579
Marital status	0.051	0.358	0.020	1.052	0.888	0.522-2.121
Reproductive history	-0.513	0.335	2.347	0.599	0.126	0.310-1.154
Medical insurance	1.572	1.085	2.100	4.817	0.147	0.574-40.394
Personal history of psychiatric disorders	1.790	0.789	5.154	5.991	0.023^*^	1.277-28.101
Comorbidities	1.199	0.601	3.986	3.318	0.046^*^	1.022-10.769
Physical activity	0.020	0.401	0.002	1.020	0.961	0.464-2.240
Place of residence	-0.092	0.280	0.107	0.912	0.743	0.527-1.579
Smoking	0.655	0.919	0.509	1.926	0.476	0.318-11.657
Alcohol consumption	-0.275	0.738	0.139	0.760	0.709	0.179-3.224
Negative life events (in the past week)	0.084	0.619	0.018	1.088	0.892	0.323-3.660
Surgical history	0.984	0.231	18.195	2.676	<0.001^*^	1.702-4.207
Pathologic diagnosis	0.263	0.562	0.219	1.301	0.639	0.433-3.910
Tumor stage	1.385	0.369	14.095	3.995	<0.001^*^	1.939-8.232
Cumulative number of chemotherapy cycles	0.551	0.210	6.874	1.734	0.009^*^	1.149-2.617
KPS score	-0.229	0.076	9.044	0.795	0.003^*^	0.685-0.923
SSRS - Objective Support	-0.112	0.028	15.722	0.894	<0.001^*^	0.846-0.945
SSRS- Subjective Support	-0.082	0.024	11.906	0.921	0.001^*^	0.879-0.965
SSRS- Utilization of Support	-0.181	0.050	13.073	0.834	<0.001^*^	0.756-0.920
RS-SC score	-0.068	0.009	64.725	0.934	<0.001^*^	0.918-0.950

^*^ *P* < 0.05.

**Figure 1:**
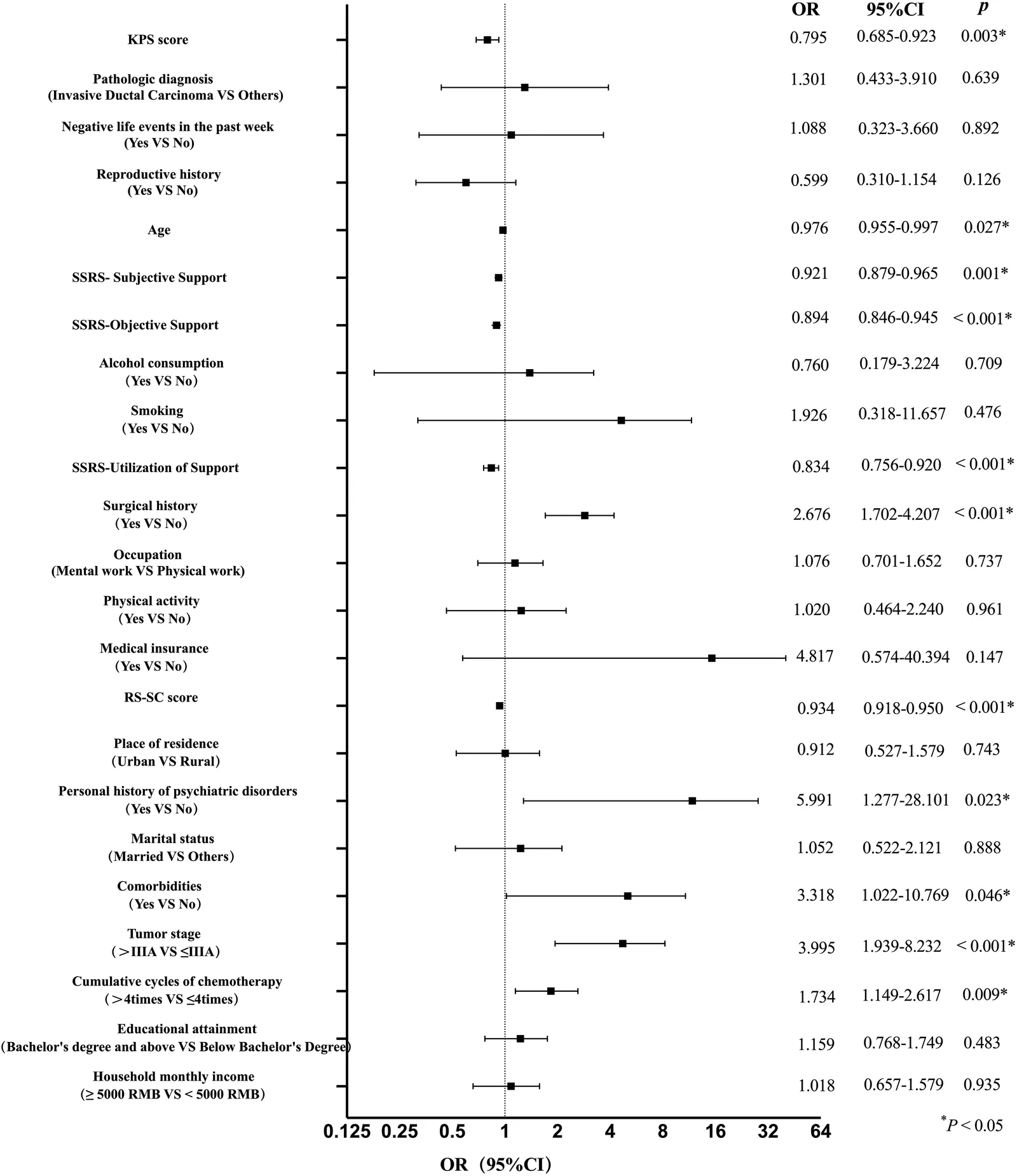
One-way logistic regression analysis of associated factors for clinically significant anxiety symptoms during chemotherapy for breast cancer.

#### 3.4.2 Multivariate logistic regression analysis

Multivariate binary logistic regression analysis was performed to identify factors associated with clinically significant anxiety symptoms among breast cancer patients during chemotherapy. Tumor stage and cumulative chemotherapy cycles were factors associated with higher odds of clinically significant anxiety symptoms, while age, KPS score, and psychological resilience were factors associated with lower odds of clinically significant anxiety symptoms (all *P* < 0.05).

Specifically, patients with tumor stage > IIIA exhibited 3.359-fold higher odds of clinically significant anxiety symptoms compared with those with stage ≤ IIIA. Similarly, patients who received more than four chemotherapy cycles had a 1.802-fold higher odds of clinically significant anxiety symptoms than those with ≤ 4 cycles.

By contrast, older age, higher KPS score, and greater psychological resilience were independently linked to lower odds of clinically significant anxiety symptoms during chemotherapy (**Table 5**; **Figure 2**).

**Table 5:** Multifactorial logistic regression analysis of associated factors for clinically significant anxiety symptoms during chemotherapy for breast cancer.

Evaluation content	*Beta*	*SE*	*Wald*	*OR*	*P*	95%*CI*
Age	-0.036	0.014	7.108	0.965	0.008^*^	0.939-0.990
Personal history of psychiatric disorders	1.404	0.879	2.551	4.070	0.110	0.727-22.787
Comorbidities	0.844	0.708	1.421	2.326	0.233	0.580-9.323
Surgical history	0.534	0.280	3.639	1.706	0.056	0.985-2.953
Tumor stage	1.212	0.459	6.982	3.359	0.008^*^	1.367-8.252
Cumulative number of chemotherapy cycles	0.589	0.252	5.474	1.802	0.019^*^	1.100-2.950
KPS score	-0.202	0.081	6.251	0.817	0.012^*^	0.697-0.957
SSRS - Objective Support	0.020	0.041	0.230	1.020	0.632	0.941-1.106
SSRS - Subjective Support	0.041	0.036	1.324	1.042	0.250	0.971-1.118
SSRS - Utilization of Support	0.072	0.081	0.784	1.074	0.376	0.917-1.259
RS-SC score	-0.080	0.011	49.576	0.923	<0.001^*^	0.903-0.944

^*^ *P* < 0.05.

**Figure 2:**
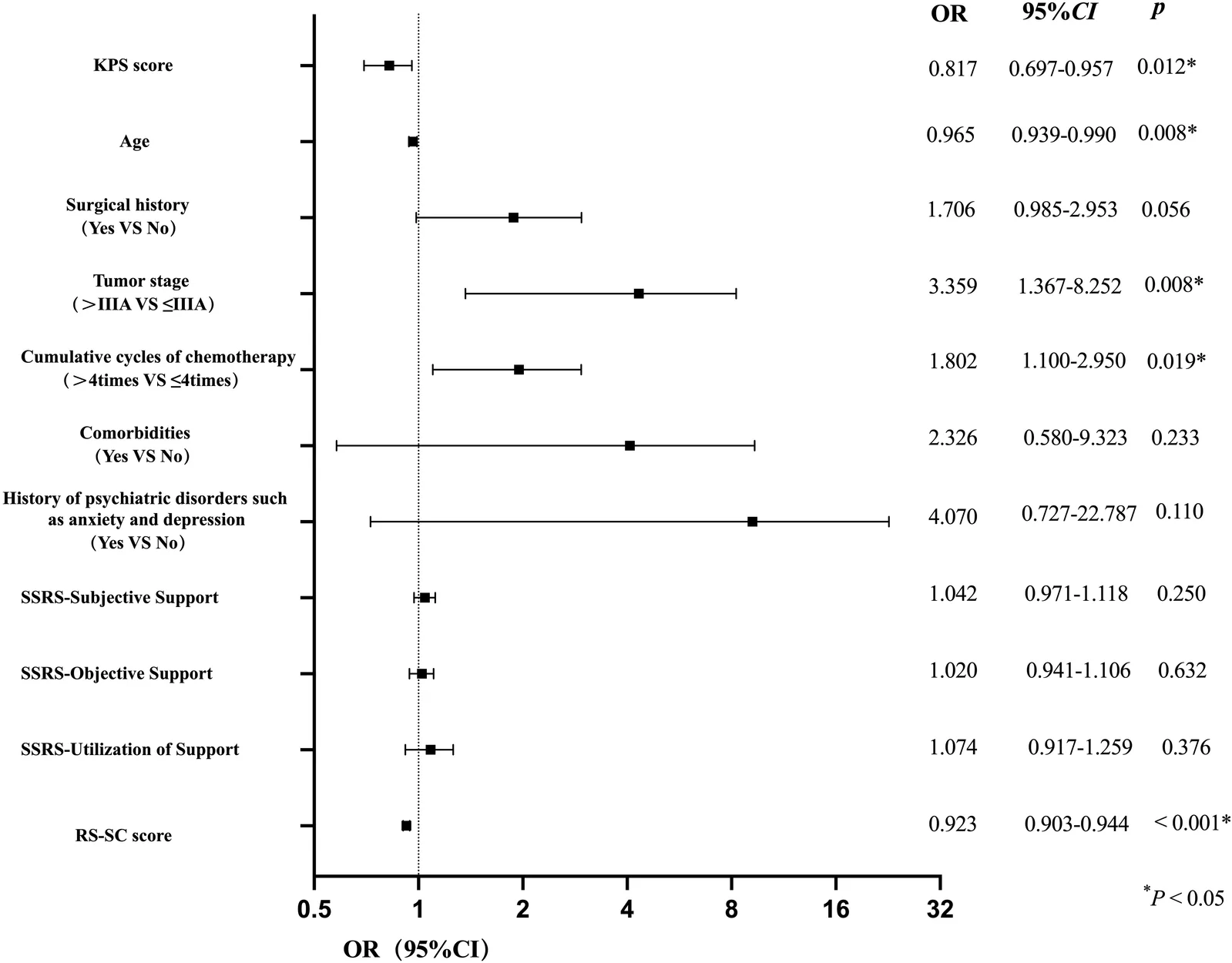
Multifactorial logistic regression analysis of associated factors for clinically significant anxiety symptoms during chemotherapy for breast cancer.

Multicollinearity assessment indicated that VIF values for predictors in the multivariable model ranged from 1.030 to 2.083, all below 5, suggesting no obvious multicollinearity issue. Model diagnostics confirmed good statistical performance of the final regression model. The Hosmer-Lemeshow test indicated acceptable model fit (χ² = 10.437, *P* = 0.236). The Nagelkerke pseudo-R² was 0.383, indicating acceptable explanatory power for the observed data.

### 3.5 Correlation analysis between high-frequency psychosomatic distress and clinically significant anxiety symptoms

Spearman’s rank correlation analysis demonstrated that clinically significant anxiety symptoms were positively correlated with difficulty initiating sleep (*ρ* = 0.358, *P* < 0.01), fatigue (*ρ* = 0.322, *P* < 0.01), and nausea (*ρ* = 0.366, *P* < 0.01) (**Figure 3**).

**Figure 3:**
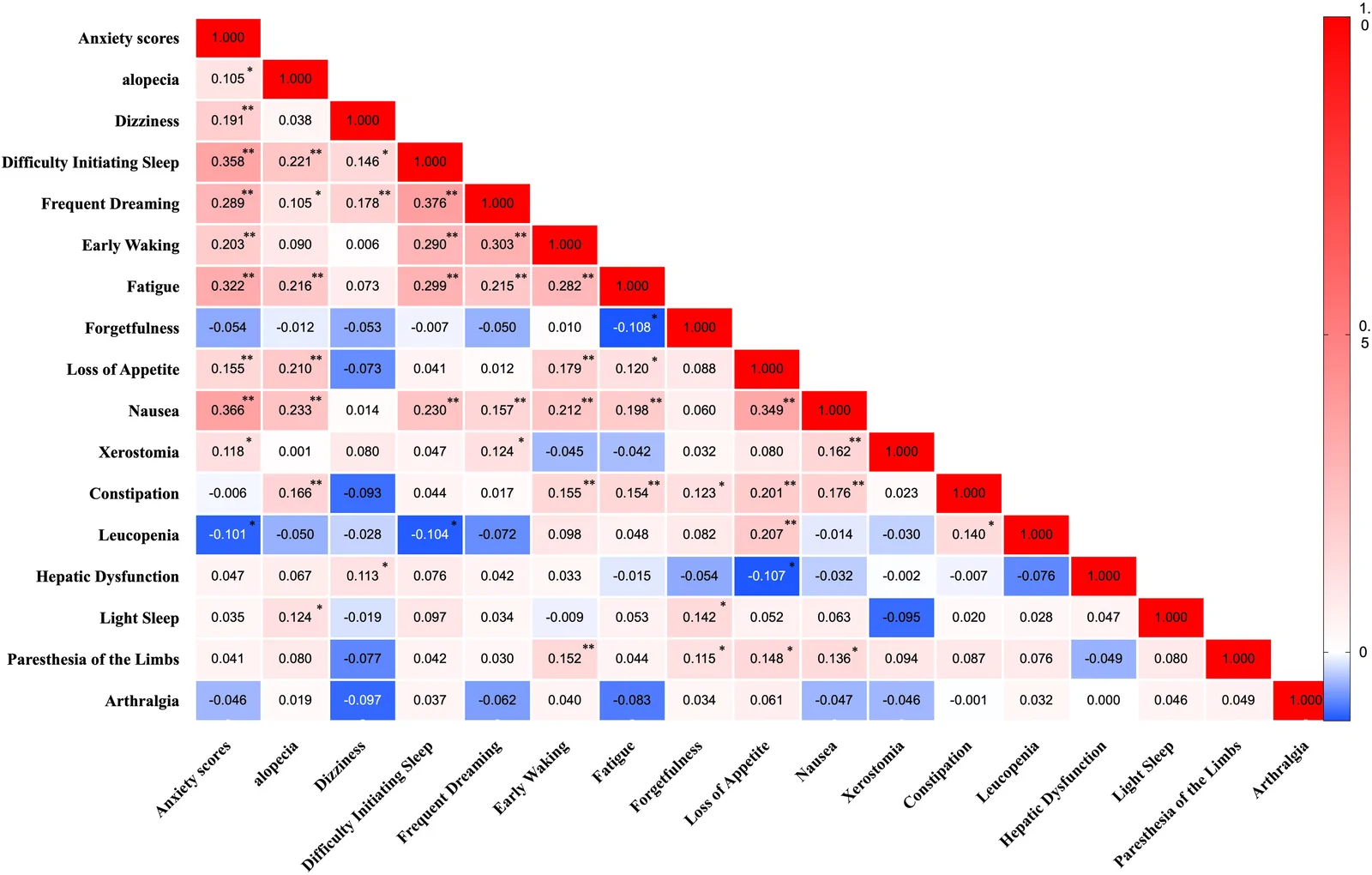
Spearman’s rank correlation coefficient matrix for high-frequency psychosomatic symptoms and clinically significant anxiety symptoms during chemotherapy for breast cancer. ^*^P < 0.05, ^**^P < 0.01.

## 4 Discussion

Breast cancer has now entered the era of chronic disease management (17). Chemotherapy, as one of the main treatment methods, causes side effects and psychological and psychiatric problems, making it difficult for patients to obtain a high quality of survival. Paying attention to patients’ psychological and psychiatric factors has become the key to the current chronic disease management of breast cancer. Under the modern medicine “biopsychosocial model” (8), identifying psychological correlates in breast cancer patients helps to improve the clinical “net benefit”.

This multicenter study enrolled 391 breast cancer patients undergoing chemotherapy from four hospitals in three regions of China. The mean age of the participants was 52.19 ± 9.54 years, and the population characteristics were consistent with the basic epidemiological features of breast cancer in China (18). The incidence of clinically significant anxiety symptoms was 43.99%. Baseline characteristic analysis of participants across centers indicated intergroup differences in variables such as subjective social support, utilization of support, and cumulative chemotherapy cycles. Although all participating centers strictly followed the NCCN guidelines for adjuvant chemotherapy of breast cancer, real world heterogeneity still existed among medical institutions in different regions, manifested in aspects such as regional sociocultural backgrounds, clinical diagnosis and treatment patterns, and psychosocial support care conditions. These differences are inherent characteristics of the population in multicenter observational studies. Notably, the primary outcome of clinically significant anxiety symptoms and key psychological variables remained balanced across centers. To control for confounding caused by baseline intergroup imbalances, this study incorporated covariates with intercenter differences along with other clinically meaningful variables into a multivariate logistic regression model, in order to adjust as much as possible for the interference of baseline differences on the estimation of independent association effects of each variable.

Multivariate logistic regression results indicated that age, psychological resilience, and KPS functional status score were factors associated with lower levels of anxiety symptoms, while advanced tumor stage and a higher number of cumulative chemotherapy cycles were positively associated with clinically significant anxiety symptoms. Univariate analysis showed that various dimensions of social support, history of psychiatric illness, comorbidities, and surgical history were correlated with clinically significant anxiety symptoms, but these associations were no longer significant in the multivariate model after adjusting for confounders. These results suggest that the buffering effect of social support on anxiety is not entirely independent; social support may exert its psychological protective effect indirectly by enhancing individual psychological resilience and maintaining physical functional status. When psychological resilience, KPS physical status, and tumor stage were simultaneously included in the regression model, the direct association contribution of social support to anxiety symptoms diminished, suggesting that in this study population, social support was not an independent correlate of clinically significant anxiety symptoms.

The above associations can be interpreted from a clinical and practical perspective. In terms of age, relatively young women with breast cancer often bear multiple pressures simultaneously, including career disruption, concerns related to fertility, and heavy family caregiving responsibilities. The stress from multiple life roles amplifies the psychological impact of cancer diagnosis. Regarding physical function, a lower KPS score indicates impaired physical function. Decreased physical strength weakens an individual’s self-efficacy and autonomy in daily life, making patients more susceptible to perceiving distress during chemotherapy. Patients with advanced tumor stage not only bear a heavier tumor burden, but also face uncertainty regarding disease recurrence and progression over the long term, which further exacerbates psychological distress. With the accumulation of chemotherapy cycles, patients repeatedly experience physical toxicities from chemotherapeutic agents, and each treatment session reawakens concerns about the disease, thereby increasing the risk of anxiety. In contrast, individuals with higher psychological resilience can reframe cancer related adversities and adopt adaptive coping strategies under treatment pressure. It should be emphasized that psychological resilience in cancer patients is not a fixed personality trait, but rather a dynamic psychological adaptive capacity that can be modulated through clinical supportive interventions (19). For chemotherapy patients, resilience oriented psychological interventions are not aimed at completely eliminating negative emotions, but rather at helping patients reconstruct disease cognition, adjust threat appraisal, and optimize coping strategies (20). Implementable intervention formats include specialized psychological interventions, tumor specific psychoeducation, and peer support integrated into routine clinical care (21, 22). However, the current evidence from resilience related intervention studies in breast cancer patients undergoing chemotherapy remains relatively limited. In the future, more clinical trials are needed to validate the actual effects of these interventions in real world clinical settings.

This study compared the results with global studies on anxiety in breast cancer over the past five years. Some previous studies suggested that older age was a correlate of anxiety in breast cancer patients (23), but more independent studies found that mental health impairment was more prominent in younger women (24–28), which is consistent with the positive association between younger age and anxiety symptoms observed in this study. Most previous literature also confirmed that advanced tumor stage was an important correlate of anxiety in breast cancer patients (24, 27), and this conclusion was also corroborated in the present study. Some studies reported a significant correlation between comorbidities and anxiety (28–30), but this association was no longer significant after multivariate adjustment in our study. Overall, some of the core findings of this study are mutually corroborated with previous literature.

Some literature has proposed that rural residence is a correlate of anxiety in breast cancer patients (23, 28, 29), but this association was not observed in the present study. A speculative explanation is that the continuous improvement of the healthcare security system has narrowed the disparities in mental health between urban and rural breast cancer patients; however, this explanation is merely a hypothesis and cannot be confirmed based on the cross sectional data of this study. In addition, marital status (25, 31), income level (24, 26, 27), frequency of healthcare service utilization (26), educational level (27, 31, 32), and occupational status (32) have been reported as significant factors associated with increased prevalence of anxiety in cancer patients. However, similar results were not obtained in the multivariate model of this study. Potential reasons for this discrepancy include that the relatively broad categorization of demographic variables in this study may obscure association signals within variables; meanwhile, differences in population sources, healthcare settings, and assessment instruments across studies may also contribute to heterogeneity of research findings.

In addition to traditional demographic, clinical, and psychosocial factors, this study further investigated high frequency psychosomatic symptoms during chemotherapy for breast cancer. The results showed that the most prevalent distressing symptoms during chemotherapy mainly included difficulty initiating sleep, light sleep, fatigue, early waking, nausea, alopecia, frequent dreaming, loss of appetite, leukopenia, constipation, forgetfulness, xerostomia, hepatic dysfunction, paresthesia of the limbs, dizziness, and arthralgia. Correlation analysis indicated that clinically significant anxiety symptoms were positively correlated with difficulty initiating sleep, fatigue, and nausea, and these three were the somatic symptoms with relatively highest correlation coefficients, and they are also very common complaints in the chemotherapy population. However, it should be noted that the correlations between these three symptoms and anxiety were only weak, indicating that the explanatory power of a single somatic symptom for anxiety outcomes is limited. Anxiety in cancer patients is affected by multiple factors including biology, psychology, treatment, and environment, so somatic symptoms are unlikely to show strong correlations; this phenomenon is relatively common in real world psycho oncology research. Even though the effect sizes are weak, when weak correlations can form an evidence chain together with clinical experience and previous literature, they still possess clinical reference value. Certainly, the actual clinical decision making value of such associations still awaits further validation by prospective cohort and intervention trials. These weak effect sizes reflect the limited but real contribution of individual somatic symptoms within a multifactorial complex system, and should not be overlooked merely because of their weak correlations. Moreover, this study adopted a cross sectional design with concurrent data collection; somatic symptoms and psychological distress were assessed simultaneously, so it can only reflect co-occurrence and cannot determine causality: worsening somatic symptoms may impair functional status, amplify threat perception, and induce psychological distress; conversely, elevated anxiety may also alter interoceptive processing, making patients more sensitive to physical discomfort and leading to more severe subjective symptom reports. This bidirectional co-occurrence suggests that clinical assessment during the chemotherapy phase should not separate physical symptom screening from psychological and emotional evaluation.

Furthermore, the screening and identification of high frequency symptoms are not simply symptom categorization; their core value lies in revealing the interaction between symptoms and disease progression and the pathological mechanisms of symptom co-occurrence. Sleep disorders can disrupt the functional homeostasis of the hypothalamic pituitary adrenal axis, disturb the balance of the central nervous system, thereby weakening individual psychological resilience and exacerbating the subjective perception of physical discomfort (33). Fatigue related to chemotherapy is also closely associated with dysregulation of the hypothalamic pituitary adrenal axis and disturbance of the central serotonin system (34). Nausea, as a common gastrointestinal adverse reaction to chemotherapy, on one hand results from the direct stimulation of the digestive tract mucosa and chemoreceptors by emetogenic chemotherapeutic agents such as anthracyclines, cyclophosphamide, and platinum compounds (35); on the other hand, psychosocial factors also play an important role. Anticipatory nausea and vomiting have been shown to be regulated by neuropsychological processes and may even affect patients’ treatment adherence (36).

The aforementioned literature provides pathophysiological support for the coexistence of difficulty initiating sleep, fatigue, nausea, and anxiety observed in this study, suggesting that these symptoms may share some neuroimmune and endocrine mechanisms. From a clinical practice perspective, systematic identification of such highly prevalent psychosomatic comorbidity symptoms may help clinicians shift from single symptom targeted management to holistic, multidimensional supportive care. Timely simultaneous screening of these somatic complaints coexisting with anxiety may help alleviate the potential self-reinforcing vicious cycle between physical burden and psychological distress.

Finally, this study still has several limitations that cannot be ignored. As a cross-sectional study, all data were derived from a single time point survey, lacking dynamic longitudinal follow up throughout the entire chemotherapy course. Therefore, it could not capture the dynamic changes of each indicator along with the treatment process, nor determine the temporal relationships among variables, so it can only analyze correlations and cannot infer causality. All outcomes and symptoms relied on patient self-report questionnaires, inevitably introducing reporting bias. Although multivariate regression adjusted for multiple known confounding factors, residual confounding still existed. In particular, this study did not collect information on specific chemotherapy regimens, drug dosages, or concurrent symptomatic supportive medications, all of which could affect somatic symptom burden and psychological status, representing unmeasured confounding in this study.

Although a multicenter design was adopted, participants were only recruited from four hospitals in three regions of China, limiting the external generalizability of the findings. During study implementation, all centers used a unified protocol, uniform inclusion and exclusion criteria, and standardized scale assessments, and all participants received adjuvant chemotherapy recommended by the NCCN guidelines, aiming to control center heterogeneity in treatment as much as possible. However, unmeasured center specific factors such as local clinical workflows, doctor patient communication patterns, and in hospital psychological care conditions could not be completely ruled out and might still confound patient reported outcomes. Therefore, this sample carries a risk of sampling bias, limiting the extrapolation of results. Future larger scale multicenter cohorts, prospective longitudinal studies, and intervention trials are needed to further validate the findings of this study.

## 5 Conclusion

The coexistence of somatic symptoms such as difficulty initiating sleep, fatigue, and nausea with anxiety suggests that comprehensive care strategies integrating somatic symptom management and psychological distress intervention are needed in clinical practice. The study sample was derived from four hospitals in China, limiting the generalizability of the findings. Constrained by the cross-sectional study design, all reported associations in this study are correlational and do not represent causality. Future prospective studies are still needed to validate the actual effects of targeted intervention strategies.

## Data Availability

The raw data supporting the conclusions of this article will be made available by the authors, without undue reservation.
