# Supplementary material for: Psychosomatic symptom profiles and associated factors of clinically significant anxiety symptoms among breast cancer patients receiving chemotherapy: a multicenter cross-sectional study in China
Source: Front Oncol. 2026 Sep 21;16:1858680. doi: 10.3389/fonc.2026.1858680 (PMC13634829; doi:10.3389/fonc.2026.1858680)
Supplement: Supplementary file 1 [file Table1.docx]

**Supplementary Table: Patient Characteristics in the Multicenter Study**

| **Evaluation**  **content** | **Overall characteristic**  **( *n* = 391)** | **Xiamen Hospital of Traditional Chinese Medicine**  **( *n* = 109)** | **Chongqing Hospital of Traditional Chinese Medicine**  **( *n* = 151)** | **The First Affiliated Hospital of Hunan University of Chinese Medicine**  **( *n* = 61)** | **Shaoyang Central Hospital**  **( *n* = 70)** | ***p*** |
| --- | --- | --- | --- | --- | --- | --- |
| Age( mean ± SD, year) | 52.19 ± 9.54 | 53.64 ± 10.70 | 51.56 ± 9.32 | 51.13 ± 8.13 | 52.23 ± 9.15 | 0.269 |
| Occupation ( *n*, [%])  Mental work  Physical work | 262 (67.01)  129 (32.99) | 71 (65.14)  38 (24.86) | 97 (64.24)  54 (35.76) | 45 (73.77)  16 (26.23) | 49 (70)  21 (30) | 0.524 |
| Educational attainment ( *n*, [%])  Bachelor's degree and above  Below bachelor's degree | 147 (37.60)  244 (62.40) | 49 (44.95)  60 (55.05) | 46 (30.46)  105 (69.54) | 26 (42.62)  35 (57.38) | 26 (37.14)  44 (62.86) | 0.092 |
| Household monthly income ( *n*, [%])  ≥ 5000 CNY  < 5000 CNY | 221 (56.52)  170 (43.48) | 65 (59.63)  44 (40.37) | 77 (50.99)  74 (49.01) | 34 (55.74)  27 (44.26) | 45 (64.29)  25 (35.71) | 0.257 |
| Marital status ( *n*, [%])  Married  Others | 356 (91.05)  35 (8.95) | 94 (86.24)  15 (13.76) | 143 (94.70)  8 (5.30) | 56 (91.80)  5 (8.20) | 63 (90)  7 (10) | 0.127 |
| Reproductive history ( *n*, [%])  Yes  No | 349 (89.26)  42 (10.74) | 95 (87.16)  14 (12.84) | 140 (92.72)  11 (7.28) | 55 (90.16)  6 (9.84) | 59 (84.29)  11 (15.71) | 0.127 |
| Medical insurance ( *n*, [%])  Yes  No | 383 (97.96)  8 (2.04) | 108 (99.08)  1 (0.92) | 148 (98.01)  3 (1.99) | 58 (95.08)  3 (4.92) | 69 (98.57)  1 (1.43) | 0.342 |
| History of psychiatric disorders such as anxiety and depression ( *n*, [%])  No  Yes | 381 (97.44)  10 (2.56) | 107 (98.17)  2 (1.83) | 148 (98.01)  3 (1.99) | 59 (96.72)  2 (3.28) | 67 (95.71)  3 (4.29) | 0.707 |
| Comorbidities ( *n*, [%])  No  Yes | 377 (96.42)  14 (3.58) | 103 (94.50)  6 (5.50) | 147 (97.35)  4 (2.65) | 58 (95.08)  3 (4.92) | 69 (98.57)  1 (1.43) | 0.423 |
| Physical activity ( *n*, [%])  No  Yes | 374 (95.65)  17 (4.35) | 100 (91.74)  9 (8.26) | 147 (97.35)  4 (2.65) | 59 (96.72)  2 (3.28) | 68 (97.14)  2 (2.86) | 0.133 |
| Place of residence ( *n*, [%])  Urban  Rural | 326 (83.38)  65 (16.62) | 99 (90.83)  10 (9.17) | 119 (78.81)  32 (21.19) | 52 (85.25)  9 (14.75) | 56 (80)  14 (20) | 0.061 |
| Smoking ( *n*, [%])  No  Yes | 386 (98.72)  5 (1.28) | 106 (97.24)  3 (2.76) | 150 (99.34)  1 (0.66) | 60 (98.36)  1 (1.64) | 70 (100)  0 (0) | 0.348 |
| Alcohol consumption ( *n*, [%])  No  Yes | 383 (97.95)  8 (2.05) | 104 (95.41)  5 (4.59) | 148 (98.01)  3 (1.99) | 61 (100)  0 (0) | 70 (100)  0 (0) | 0.100 |
| Negative life events (in the past week) ( *n*, [%])  Yes  No | 75 (19.18)  316 (80.82) | 20 (18.35)  89 (81.65) | 25 (16.56)  126 (83.44) | 18 (30)  43 (70) | 12 (20)  58 (80) | 0.164 |
| Surgical history ( *n*, [%])  No  Yes | 83 (21.23)  308 (78.77) | 30 (27.52)  79 (72.48) | 33 (21.85)  118 (78.15) | 10 (16.39)  51 (83.61) | 10 (14.29)  60 (85.71) | 0.139 |
| Pathologic diagnosis ( *n*, [%])  Invasive Ductal Carcinoma  Others | 355 (89.20)  36 (10.8) | 97 (88.99)  12 (11.01) | 141 (93.38)  10 (6.62) | 54 (88.52)  7 (11.48) | 63 (90)  7 (10) | 0.564 |
| Tumor stage ( *n*, [%])  ≤ⅢA  >ⅢA | 356 (91.05)  35 (8.95) | 99 (90.83)  10 (9.17) | 133 (88.08)  18 (11.92) | 56 (91.80)  5 (8.20) | 68 (97.14)  2 (2.86) | 0.181 |
| Cumulative cycles of chemotherapy ( *n*, [%])  ≤ 4 times  > 4 times | 340 (86.96)  51 (13.04) | 101 (92.66)  8 (7.34) | 119 (78.81)  32 (21.19) | 58 (95.08)  3 (4.92) | 62 (88.57)  8 (11.43) | 0.001* |
| KPS score( mean ± SD) | 89.57 ± 2.04 | 89.45 ± 2.29 | 89.67 ± 1.80 | 89.51 ± 2.18 | 89.57 ± 2.04 | 0.854 |
| SSRS-Objective Support ( mean ± SD) | 12.72 ± 3.73 | 12.38 ± 4.12 | 12.63 ± 3.50 | 13.41 ± 3.36 | 12.83 ± 3.89 | 0.369 |
| SSRS-Subjective Support  ( mean ± SD) | 22.26 ± 4.47 | 20.99 ± 4.32 | 22.85 ± 4.72 | 23.43 ± 3.83 | 21.96 ± 4.26 | 0.001* |
| SSRS - Utilization of Support  ( mean ± SD) | 6.85 ± 2.16 | 6.72 ± 2.42 | 6.91 ± 2.08 | 7.49 ± 1.84 | 6.33 ± 2.01 | 0.018* |
| RS-SC score ( mean ± SD) | 99.08 ± 16.22 | 98.77 ± 18.04 | 97.26 ± 15.89 | 101.18 ± 13.88 | 101.66 ± 15.60 | 0.192 |
| Clinically significant anxiety symptoms( *n*, [%])  No  Yes | 219 (56.01)  172 (43.99) | 60 (55.05)  49 (44.95) | 78 (51.66)  73 (48.34) | 34 (55.74)  27 (44.26) | 47 (67.14)  23 (32.86) | 0.193 |

****p* < 0.05. Supplementary Table. Baseline characteristics of participants stratified by four multicenter sites.** Categorical variables are presented as *n* (%), continuous variables as mean ± SD. *P* values for categorical variables were obtained by χ² test where appropriate, otherwise by ***Fisher’s* exact test**. One-way ANOVA was used for continuous variables. Variables with significant inter-center differences were adjusted in multivariate logistic regression to control multicenter confounding.
